# A new image encryption algorithm based on logistic chaotic map with varying parameter

**DOI:** 10.1186/s40064-016-1959-1

**Published:** 2016-03-08

**Authors:** Lingfeng Liu, Suoxia Miao

**Affiliations:** School of software, Nanchang University, Nanchang, 330031 People’s Republic of China; Faculty of Science, Nanchang Institute of Technology, Nanchang, 330029 People’s Republic of China

**Keywords:** Chaos, Image encryption, Dynamical algorithm, Parameter-varied logistic map

## Abstract

In this paper, we proposed a new image encryption algorithm based on parameter-varied logistic chaotic map and dynamical algorithm. The parameter-varied logistic map can cure the weaknesses of logistic map and resist the phase space reconstruction attack. We use the parameter-varied logistic map to shuffle the plain image, and then use a dynamical algorithm to encrypt the image. We carry out several experiments, including Histogram analysis, information entropy analysis, sensitivity analysis, key space analysis, correlation analysis and computational complexity to evaluate its performances. The experiment results show that this algorithm is with high security and can be competitive for image encryption.

## Background

With the rapid development in internet technology and multimedia technology, multimedia communication has become more and more important. Therefore, image encryption has become an increasingly serious issue and urgently needed (Ye and Wong 2013). However, traditional encryption algorithms, such as RSA, DES and IDEA, are not suitable for image encryption due to image’s intrinsic properties such as bulky data capacity, strong redundancy and strong correlations among adjacent pixels (Wang et al. 2012; Chen et al. 2013; Coppersmith 1994).

Chaotic system has many important properties, such as unpredictability, similar randomness, aperiodicity, sensitive dependence on initial conditions and parameters, these properties make chaotic systems become popular in image encryption (Huang 2012; Wang and Guo 2014; Zhang and Zhao 2014; Zhang and Liu 2011; Hua et al. 2015; Tong et al. 2015; Hussain and Shah 2013). Among all the chaotic encryption image algorithm, the low-dimensional chaotic map are always used for its easily implement, such as logistic map. However, some common weaknesses of the logistic map, including relatively small key space and uneven distribution of sequences, et al, bring some security risks for encryption. On the other hand, for a deterministic chaotic system, the chaos behaviors can be discerned by using some methods in chaos theory. Once we find some information about the chaotic system, we can use such information to help us finding the secret key. In many chaotic ciphers, such as Kanso and Smaoui (2009), Zhou and Liao (2012), Sun et al. (2010), Pareek et al. (2005), Wong et al. (2003), Liu and Wang (2010), Wang et al. (2012), Patidar et al. (2010), Gonzalez and Hernandez (2013), the ciphertext directly depends on the chaotic orbit of a single chaotic system, the orbit sequence comes to be stationary, so the extraction of such information may be possible by using some chaos theory methods such as phase space reconstruction. In Short (1994), short use the phase space reconstruction method, has successfully attacked almost all the low-dimensional chaotic systems. Wang and Luan (2013) propose a three-dimensional coupled logistic maps to overcome the weaknesses of logistic map, however, the system is still deterministic, and is still under the risk of being attacked by phase space reconstruction.

As we know, varying the parameters can disrupt the phase space of a chaotic system, and improve the security to resist the phase space reconstruction attack. Some varying parameter techniques have been proposed, e.g., Murillo-Escobar et al. (2015) use 32 hexadecimal digits to vary the parameter and initial value of logistic map, and the proposed system can avoid the small key space of low dimensional chaotic systems. This varying technique is given by 32 fixed hexadecimal digits, which is not that secure. Using a prediction technique based on wavelet neural network and multiwavelets neural network can predict the parameter-varying chaotic system whose parameters are varying in a simple way (Xiao and Gao 2006). Wang et al. (2009) use the generated sequences by logistic map to control three kinds of typical two-dimensional chaotic maps, but do not show the performances of their parameter-varied chaotic maps.

Therefore, in order to improve the weaknesses of logistic map and resist the phase space reconstruction attack, we propose an image encryption algorithm based on logistic map with varying parameter. The varying technique is based on the zero-mean logistic map, which can make the parameter varying in a random-like way. We show that the parameter-varied logistic map can cure the weaknesses of logistic map and is capable to resist phase space reconstruction. Furthermore, we use a dynamical algorithm in our encryption algorithm. Our encryption algorithm is related to the plaintext, which can resist known and chosen-plaintext attacks. The experimental results show that the proposed algorithm is with high security, and can be competitive to other proposed algorithms.

The rest of this paper is organized as follows. In “Shuffling algorithm” section, a shuffling algorithm based on parameter-varied logistic system is described. We show that the parameter-varied logistic system can cure the common weaknesses and is capable to resist phase space reconstruction. “Dynamical encryption algorithm” section introduce a dynamical algorithm for the image encryption. The experimental results, analysis and comparison are shown in “Experimental analysis” section. Finally, “Conclusion” section concludes the paper.

## Shuffling algorithm

In this section, we propose a shuffling method based on parameter-varied chaotic map. Perhaps, the one-dimensional maps are the simplest mathematical objects to display chaotic behavior (Lasota and Mackey 1994). The logistic maps are one kind of one-dimensional maps, which were described in May (1976) and have already been widely used in image encryption

1 $$x_{i + 1} = f(x_{i} ) = ax_{i} (1 - x_{i} )$$

here, *a* is the parameter of logistic map, *x*_*i*_ = *f*^(*i*)^(x_0_) ∈ *I*, *i* = 0, 1, 2,… and *f*: *I* → *I*, where *I* denotes an interval. For 3.5699 < *a* ≤ 4, Eq. (1) turns to be chaotic. Using this function, we can obtain a real-valued sequence by iteration of an initial value *x*_0_. Since some researches show that the sequences generated by logistic map are not secure with some weaknesses (Wang and Luan 2013), including relatively small key space, an uneven distribution and easily be attacked by phase space reconstruction, therefore, we use the following parameter-varying logistic map in our algorithm.

2 $$x_{i + 1} = f_{k} (x_{i} ) = (10^{6} - 1) \cdot a_{k} x_{i} (1 - x_{i} )\hbox{ mod }1, \, k = 1,2, \ldots ,M$$

here, *a*_*k*_ is the varied parameter, *M* is the cardinality of the parameter set. We use the following zero-mean logistic map to vary the parameter *a*_*k*_

3 $$u_{k + 1} = 1 - 2u_{k}^{2} \, ( - 1 \le u_{k} \le 1)$$

Divide the interval [−1, 1] into *M* sub-intervals *τ*_*i*_, *i* = 0, 1, …, *M* − 1. Denote *τ*_*i*_ = [*t*_*i*_, *t*_*i*+1_), *i* = 0, 1, …, *M* − 2, and *τ*_*M*−1_ = [*t*_*M−*1_, *t*_*M*_], where

4 $$t_{i} = - \cos \left( {\frac{k}{M}\pi } \right)$$

Then, *α* = {*τ*_0_, *τ*_1_, …, *τ*_*M*−1_} is a finite measurable partition of *I*. Denote a correspondence *S*: *I* → {0, 1, 2, …, *M* − 1} from the set *I* to the set {0, 1, 2, …, *M* − 1}. For any *u*_*k*_, define

5 $$s(u_{k} ) = i,\,\,{\text{ if }}u_{k} \in \tau_{i}$$

here *s*(*u*_*k*_) is the symbol representation of the real number *u*_*k*_ according to the partition *α*. Then, the generated integer sequence is denoted as {*s*_*k*_} and can be proved to be uniformly distributed in set {0, 1,…, *M* - 1} (Hu et al. 2004). Let the parameter set be {*c*_1_, *c*_2_,…, *c*_*M*_}, we use the sequence {*s*_*k*_} to vary the parameter *a* as

6 $$a_{k} = c_{{s_{k} + 1}}$$

Then, the parameter *a*_*k*_ of Eq. (2) is varying chaotic in the set {*c*_1_, *c*_2_,…, *c*_*M*_}. Let *n* be the steps of iteration with each parameter *a*_*k*_, we can generate the chaotic binary sequences by using the following algorithm.

7 $$b_{i} = \left\{ \begin{aligned} 0, \quad x_{i} \le 0.5 \hfill \\ 1, \quad x_{i} > 0.5 \hfill \\ \end{aligned} \right. \,\,\, i = 0,1, \ldots$$

**Fig. 1:**
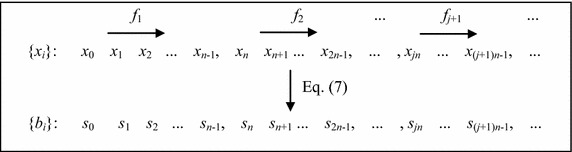
The output of logistic map with varying parameter

**Fig. 2:**
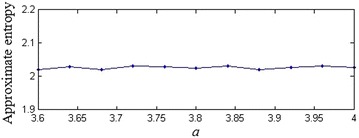
The ApEns of logistic map with different parameters

**Fig. 3:**
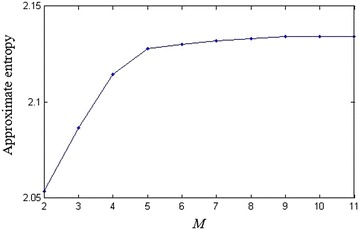
The ApEns of logistic map with different *M*

Figure 1 shows the main frame of our pseudorandom bit generator. As we seen, the number *M* of different values of the parameter and the iteration step *n* for each parameter are two important parameters in our parameter-varied logistic map. Studies show that the logistic map can be reconstructed with delay time 1 and embedding dimension 3 (Han et al. 2015). For each parameter, if we don’t generate enough data, the reconstruction will fail. Therefore, we have that *n* < 3 is more suitable. In this paper, we choose *n* = 1, which can be regarded as a well-known random logistic map. Obviously, the larger the number *M* is, the more kinds of iterative rule has. However, it is impossible to choose *M* to be infinite. In order to determine the value *M*, we use approximate entropy (ApEn) to evaluate the complexity of the generated sequences. Before this experiment, we first calculate the ApEns of sequences generated by different parameter *a*_*k*_ in Fig. 2, which indicates that the generated sequence has approximately the same complexity with different parameter. The ApEns with different *M* is shown in Fig. 3. From Fig. 3 we have that, when *M* is close to 9, the complexity approximately remains the same. As the complexity has almost no relation to the value of parameter, thus, is only influenced by the number of different parameters. Therefore, in this paper, we choose *M* = 9.

**Fig. 4:**
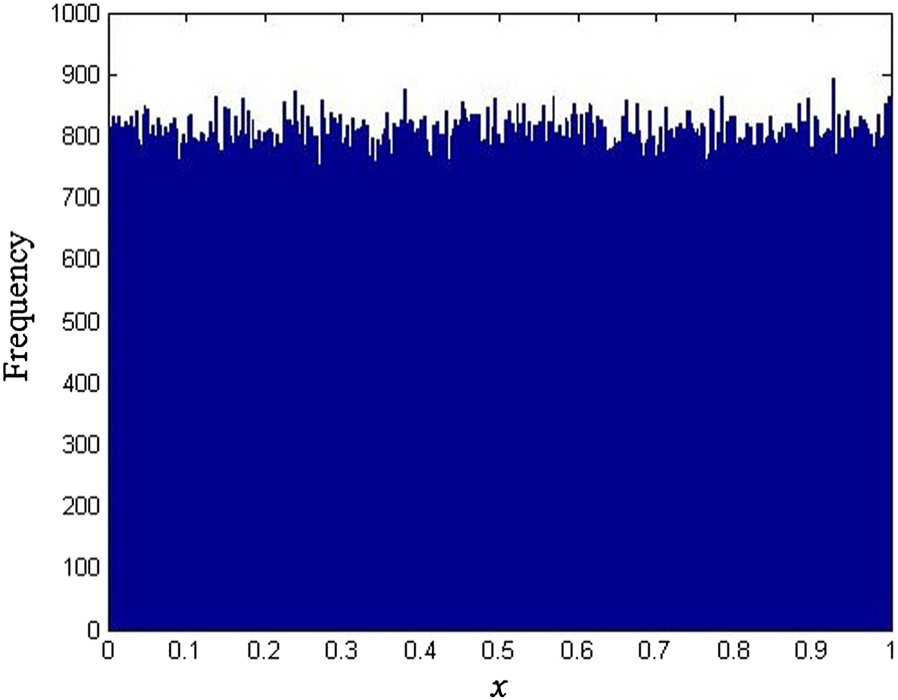
The distribution of the generated sequence

**Fig. 5:**
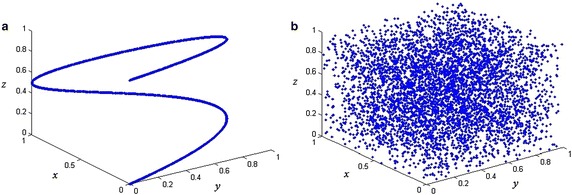
Reconstructed phase space of (**a**) logistic map (**b**) logistic map with varying parameter

Next, we show that our logistic map with varying parameter can improve the weaknesses of logistic map. Firstly, the initial values *x*_0_, *u*_0_ and nine different parameters *a*_1_,…, *a*_9_ can be selected as the secret keys, which has greatly improved the key space of logistic map. Then, we have that the distribution of the generated sequences of our logistic map with varying parameter is uniform. We take *x*_0_ = 0.1, *u*_0_ = 0.2 and *a*_*k*_ = 3.9 + 0.01*k, *k* = 1, 2,…, 9 as an example, the distribution of the generated sequence is shown in Fig. 4. Furthermore, we would show that our chaotic map can resist the phase space reconstruction. There are two key parameters in the phase space reconstruction, delay time and embedding dimension. By using auto-correlation function and false neighbor method, we have the optimal delay time be 1 and the embedding dimension be 3. We use use these two parameters to reconstruct the phase space in Fig. 5. From Fig. 5 we have that the reconstructed phase space has a significant structure of logistic map, while for the logistic map with varying parameter, the reconstructed phase space is disordered with no significant structure. Thus, the logistic map with varying parameter can resist the phase space reconstruction. Moreover, for other delay time and embedding dimension, the phase space is still disordered with no significant structure, which we do not repeat it here. Finally, we discuss the stable and unstable manifolds proposed in (Ragulskis and Navickas 2011) of our logistic map with varying parameter. For the logistic map, (Ragulskis and Navickas 2011) shows that the misplacement of the initial condition could potentially lead to the non-asymptotic convergence to a finite length periodic orbit, which makes the logistic sequence weak to be used in encryption. As (Ragulskis and Navickas 2011) shown, the initial conditions leading to the period solution in different forward iterations with different parameters are all different. Thus, our logistic map with varying parameter can naturally overcome such weakness. If the value of *x*_*i*_ falls into the set which will lead to a period solution after several iterations with fixed parameter *a*, the generated sequence will jump out from the period solution because of the varying of parameter *a*, as well as the initial conditions leading to the period solution with different parameters are different.

Now we can introduce the shuffling algorithm. Let the size of the gray image *g* is *p* × *q*, and *g*(*x*, *y*) is the value of pixel at the *x*th row and *y*th column of the plain image. Reshape the plain image into one-dimensional array *g*(*i*), *i* = 1, 2, …, *p* × *q*. By using the binary sequence {*b*_*i*_}, we can shuffle the image.

Set *L*, *R*, *Z* be three empty arrays. Begin with *i* = 1, add 1 every time, and end with *i* = *p* × *q*. If *s*_*i*_ = 1, *g*(*i*) is put into array *L* in sequence. If *b*_*i*_ = 0, *g*(*i*) is put into array *R* in sequence. Merge *L* and *R* into the array *Z*. If round *T* is odd, put *L* in front of *R*, else, put *R* in front of *L*. Finally, change the array *Z* into two-dimensional matrix *G* with *p* × *q*. Then the image *G* is the shuffled image. This method is first proposed in (Wang and Guo 2014).

## Dynamical encryption algorithm

We use the following dynamical algorithm to encrypt the shuffled image *G*. The steps are

1. *Initialization:* Denote the initial code book as follow.
  8 $$B_{0} = \left( {\begin{array}{*{20}c} 1 & 2 & \cdots & {2^{N} } \\ {b_{1} (0)} & {b_{2} (0)} & \cdots & {b_{{2^{N} }} (0)} \\ \end{array} } \right)$$
  here, *B*_0_(*i*) = *b*_*i*_(0), and {*b*_*i*_(0)} is an arbitrary permutation from 1 to 2^*N*^.
2. *Code transformation:* Consider the array *Z*(*i*), change *Z*(*i*) into binary representation, *Z*(*i*) = (*Z*_*i*1_*Z*_*i*2_*…Zi*_*N*_)_2_. Denote *q*_*i*_ = 2^*α*^ (*α* = *Z*_*i*1_*Z*_*i*2_*Z*_*i*3_), and *w*_*i*_ = 8*β (*β* = *Zi*_4_*Zi*_5_*…Zi*_*N*_). Use the following two algorithms *R*(·) and *C*(·) to transform the code book.
  9 $$R\left( q \right) = \left( {\begin{array}{*{20}c} 1 & 2 & \cdots & M \\ {k_{1} } & {k_{2} } & \cdots & {k_{M} } \\ \end{array} } \right),k_{d} = \left\{ {\begin{array}{ll} d, &\quad {d \in \left[ {1,q} \right]} \\ d + q - M/2, &\quad d \in \left[ {M/2 + 1,M/2 + q} \right] \\ {q + d}, &\quad d \in \left[ {q + 1,M/2} \right] \cup \left[ {M/2 + 1 + q,M} \right] \end{array} } \right.$$
  10 $$C\left( w \right) = \left( {\begin{array}{*{20}c} 1 & 2 & \cdots & M \\ {k_{1} } & {k_{2} } & \cdots & {k_{M} } \\ \end{array} } \right),\,\,k_{d} = \left\{ {\begin{array}{ll} {M - 1 - w + d}, & d \in \left[ {1,w + 1} \right] \hfill \\ {d - w - 1}, &\quad d \in \left[ {w + 2,M} \right] \hfill \\ \end{array} } \right.$$
  Then *B*_*i*+1_ = *B*_*i*_(*C*(*w*)*R*(*q*))^−1^.
3. *Search the code book:* For any driven element *Z*(*i*), we have *k*(*i*) = *b*_*Z*(*i*)_(*i*);
4. *Stop command:* If *i* ≠ NULL, then *i* = *i* + 1, back to step 2); Otherwise, stop the algorithm.

**Fig. 6:**
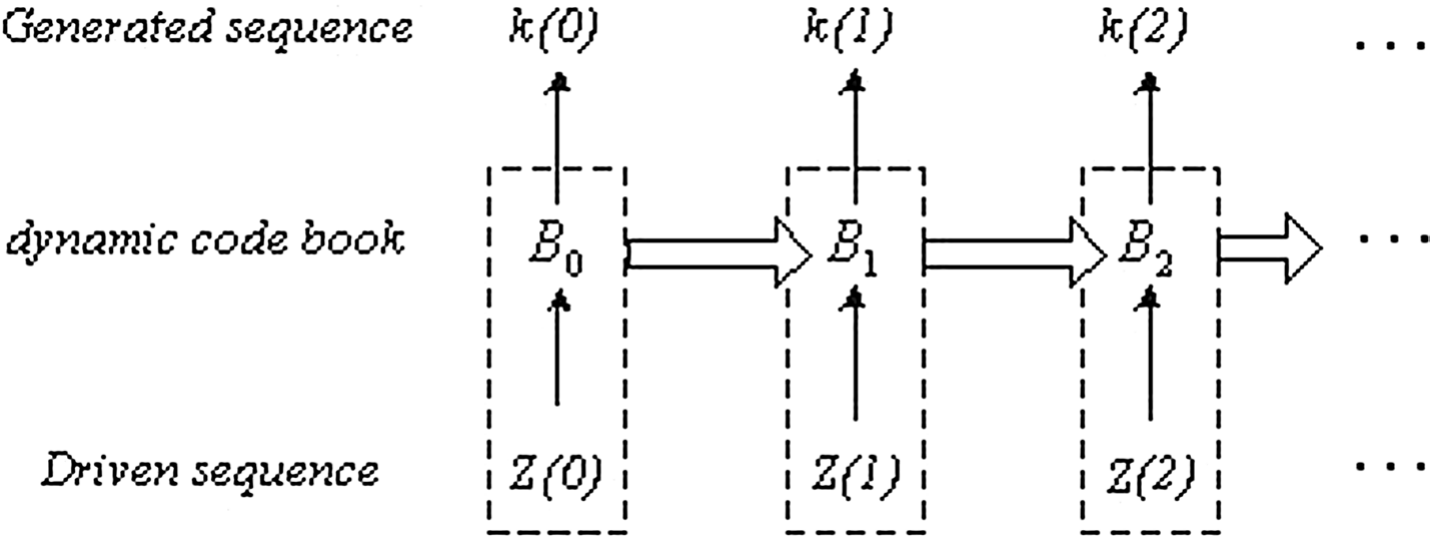
The main frame of our dynamic algorithm

Figure 6 shows the main frame of our dynamic algorithm.

The shuffled array {*Z*(*i*)} is used as the driven sequence. Change the array {*k*(*i*)} into two-dimensional matrix *G*’ by sequential scanning. The image *G*’ is the ciphered image. In this encryption algorithm, the initial values *x*_0_ and *u*_0_, different parameters *a*_1_,…, *a*_9_, and the initial code book can be selected as the secret keys. Both shuffling and dynamical encryption algorithm are reversible, thus, the decryption algorithm is just the inverse process of the encryption algorithm with using the same secret keys.

## Experimental analysis

In our experiments, we select the gray-scale image “Lena.bmp” sized 256 × 256 as the plain image. Choose key parameters *a*_*k*_ = {3.991, 3.992, 3.993, 3.994, 3.995, 3.996, 3.997, 3.998, 3.999}, *T* = 1, and the initial code book as

$$B_{0} = \left( {\begin{array}{cccc} 1 & 2 & \cdots & {2^{N} } \\ 1 & 2 & \cdots & {2^{N} } \\ \end{array} } \right).$$

**Fig. 7:**
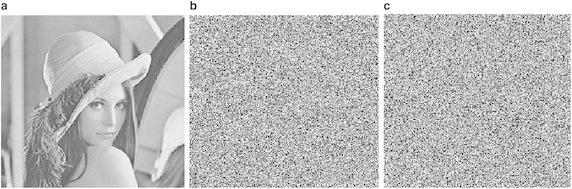
**a** Plain image, **b** Shuffled image when *T* = 2; **c** Ciphered image

Figure 7 shows the encryption effect of each step in the proposed method. Furthermore, we use several security tests to show the good performances of our algorithm.

### Histogram of the image

**Fig. 8:**
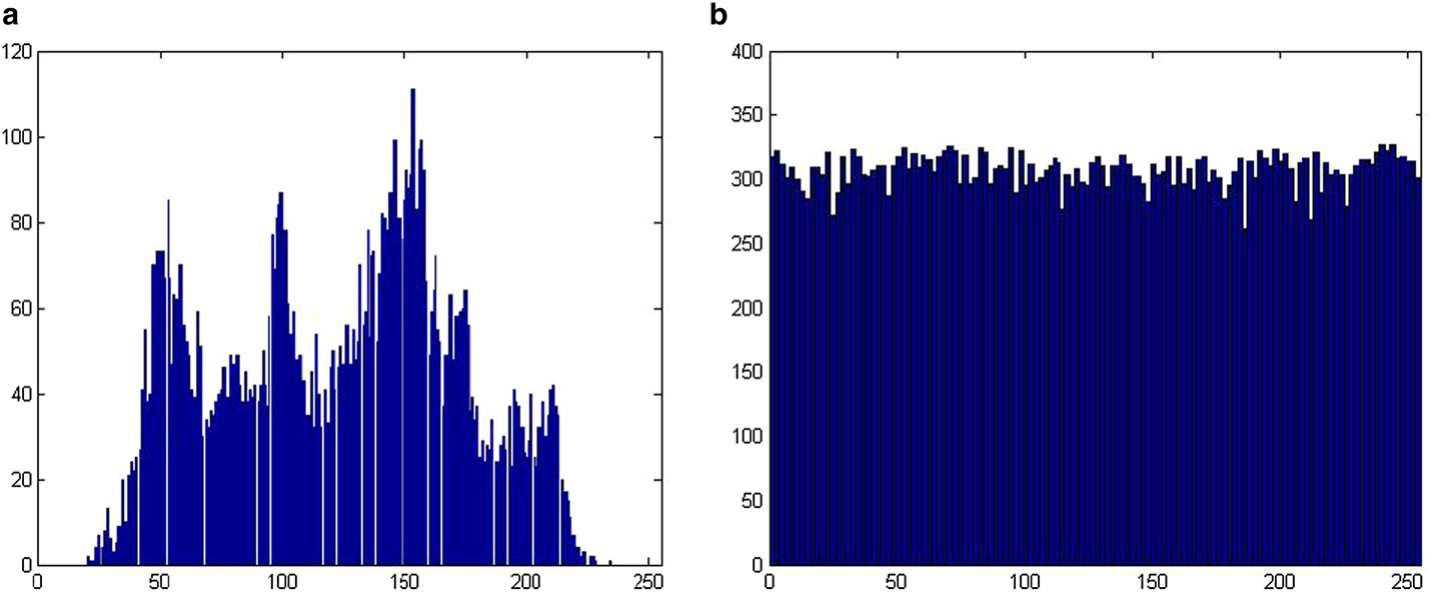
Histogram of the images **a** Plain image, **b** Ciphered image

The distribution of the ciphered image is a major concern. Here, we use the histogram to show the distribution of the plain image and the cipher image. From Fig. 8 we know that the proposed scheme results in very flat distributions of ciphered images, which can resist cipher-only attack.

### Information entropy analysis

Information entropy is the most significant measure to disorder, or unpredictability. The information entropy can be calculated as

$$H(m) = - \sum\limits_{i = 1}^{M} {p(m_{i} )\log_{2} p(m_{i} )}$$

here, *M* is the total number if symbols, and *p*(*m*_*i*_) is the probability of symbol *m*_*i*_. For a random image with 256 gray levels, *M* = 256, the entropy should ideally be 8.

The entropies of plain image and ciphered image are calculated. The results are shown in Table 1. From Table 1 we know that the entropies of the ciphered image produced by our algorithm are very close to the value of 8, which means that the ciphered images are close to a random source, and performs better than the algorithms in Wang and Guo (2014), Zhou and Liao (2012) and Sun et al. (2010).

**Table 1:** Information entropy of the ciphered images

Different algorithms	*H* (*m*)
Our algorithm with *T* = 1	7.9995
Our algorithm with *T* = 2	7.9994
Our algorithm with *T* = 3	7.9995
Ref (Wang and Guo 2014)	7.9977
Ref. (Zhou and Liao 2012)	7.9966
Ref. (Sun et al. 2010)	7.9965

### Sensitivity analysis

In order to resist differential analysis, the cipher text should be sensitive to both plain text and secret key.

#### Plaintext sensitivity

The Number of Pixels Change Rate (NPCR) and Unified Average Changing Intensity (UACI) are commonly used to evaluate the sensitivity to plain text. For two images *x* = {*x*_1_, *x*_2_, …, *x*_*n*_} and *y* = {*y*_1_, *y*_2_, …, *y*_*n*_}, the NPCR and UACI are defined as follows

$$NPCR = \frac{1}{n}\sum\limits_{i = 1}^{n} {D(x_{i} ,y_{i} )}$$

$$UACI = \frac{1}{n}\sum\limits_{i = 1}^{n} {\frac{{|x_{i} - y_{i} |}}{255}}$$

here, *D*(*x*_*i*_, *y*_*i*_) = 0 if *x*_*i*_ = *y*_*i*_ and *D*(*x*_*i*_, *y*_*i*_) = 1 if *x*_*i*_ ≠ *y*_*i*_. For the gray image, the ideal value of NPCR and UACI are 0.9961 and 0.3346, respectively.

We randomly change only 1 bit in the original plain image, and use the same secret key to encrypt the modified image and the original image. Then we calculate their NPCR and UACI values. The results are shown in Table 2.

**Table 2:** NPCR and UACI when *T* takes different values

Round *T*	NPCR	UACI
1	0.9949	0.3156
2	0.9971	0.3398
3	0.9963	0.3371

From Table 2 we find that both the NPCR and UACI value are close to the ideal value when shuffling round *T* > 1. This means that the proposed scheme can effectively resist the differential attack and chosen-plaintext attack.

#### Key sensitivity

**Fig. 9:**
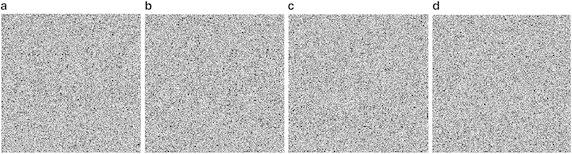
Key sensitivity analysis. **a** decrypted image with *x*_0_ be different with 10^−14^ **b** decrypted image with *u*_0_ be different with 10^−14^ **c** decrypted image with *a*_1_ be different with 10^−14^ **d** decrypted image by randomly exchanging two codes in the initial code book

We test the sensitivity to secret key using one of the keys that is a little different from the original one. As we shown, the initial values *x*_0_ and *u*_0_, different parameters *a*_1_,…, *a*_9_, and the initial code book can be used as the secret key. We decrypt the encrypted image with *x*_0_ be different with 10^−14^, the decrypted image is shown in Fig. 9a. The decrypted image with *u*_0_ and *a*_1_ be different with 10^−14^ is shown in Fig. 9b, c, respectively. Furthermore, randomly exchange two codes in the initial code book, the decrypted image is shown in Fig. 9d. From Fig. 9 we can see that all the decrypted images can not be recognized, which indicates that the secret keys are highly sensitive.

#### Key space

The key space should be large enough to withstand attacks. In our proposed encryption algorithm, the initial values *x*_0_, *u*_0_, the varied parameters *a*_*k*_ and the initial code book can be selected as secret keys. Let the largest precision be 10^−14^, the key space is about

$$10^{14} \cdot 10^{14} \cdot (0.4 \cdot 10^{14} )^{9} \cdot 256! \approx 2^{2183}$$

On the other hand, the experimental results show that our scheme is highly sensitive to the secret key. Therefore, The key space of our algorithm is much larger than 2^128^, and is also larger than 2^160^ of (Wang and Guo 2014) and 2^140^ of (Tong et al. 2015), under the same precision, which concludes that our algorithm can sufficiently resist all kinds of brute-force attacks.

#### Correlation analysis

A good image encryption algorithm should remove this strong correlation between adjacent pixels. The correlation property can be quantified by means of correlation coefficients as

$$r = \frac{{\text{cov} (x,y)}}{{\sqrt {D(x)D(y)} }}$$

where

$$\text{cov} (x,y) = \frac{1}{n}\sum\limits_{i = 1}^{n} {[x_{i} - E(x)][y_{i} - E(y)]}$$

$$D(x) = \frac{1}{n}\sum\limits_{i = 1}^{n} {[x_{i} - E(x)]^{2} }$$

$$E(x) = \frac{1}{n}\sum\limits_{i = 1}^{n} {x_{i} }$$

*x*_*i*_ and *y*_*i*_ are two adjacent pixels, *n* is the total number of adjacent pixel pairs (*x*_*i*_, *y*_*i*_). Table 3 gives the correlation coefficients of plain image and encrypted image. It is clear that all the correlation coefficients of encrypted images are close to zero, which means that our proposed algorithm can effectively remove the correlations among the adjacent pixels of the plain image, and can resist statistical attacks. Also, our algorithm performs better than the algorithms in Wang and Guo (2014), Hua et al. (2015) and Tong et al. (2015) in this sense.

**Table 3:** Correlation coefficients of the plain and ciphered images

Images	Horizontal	Vertical	Diagonal
Plain image	0.98496	0.97179	0.96853
Encrypt with *T* = 1	0.0088	0.0083	0.0121
Encrypt with *T* = 2	0.0053	0.0040	0.0062
Encrypt with *T* = 3	0.0021	0.0046	0.0033
Ref. (Wang and Guo 2014)	0.0063	0.0063	0.0069
Ref. (Hua et al. 2015)	0.0024	−0.0086	0.0402
Ref. (Tong et al. 2015)	0.0038	0.0058	0.0133

#### Computational complexity

Here, we compare the computational complexity of our algorithm with the traditional DES and AES algorithms. All the algorithms are experiment by Matlab R2014a on the computer with 3.6 GHz CPU and 8 GB memory. The test results are shown in Table 4. From Table 4 we can see that the time of our algorithm with *T* = 1 and 2 are both less than the DES and AES algorithms, and is quite acceptable for image encryption. Certainly, the larger the *T* is, the more the time needed, and more secure the algorithm is. Therefore, users can choose a suitable *T* for their different security demand.

**Table 4:** Encryption speed of each scheme

Different algorithms	Encryption time (s)
Our algorithm with *T* = 1	0.05944
Our algorithm with *T* = 2	0.06569
DES	0.59784
AES	0.11297

## Conclusions

In this paper, we propose a new image encryption algorithm based on parameter-varied chaotic map and dynamical algorithm. The varied parameters are controlled by zero-mean logistic map and hopping in the given parameter set. We show that the proposed logistic map can overcome the common weaknesses of and is capable to resist phase space reconstruction. We carry out many experiments, including Histogram analysis, information entropy analysis, sensitivity analysis, key space analysis, correlation analysis and computational complexity, to show the security and performance of the proposed image encryption scheme. The experimental results show that our algorithm is with high security, and can be competitive with some other proposed image encryption algorithms.
